# The prevalence of posterior inferior tibiofibular ligament and inferior tibiofibular transverse ligament injuries in syndesmosis-injured ankles evaluated by oblique axial magnetic resonance imaging: a retrospective study

**DOI:** 10.1186/s12891-022-05220-0

**Published:** 2022-03-18

**Authors:** Kousuke Shiwaku, Atsushi Teramoto, Kousuke Iba, Hidenori Otsubo, Tomoaki Kamiya, Hiroaki Shoji, Kota Watanabe, Toshihiko Yamashita

**Affiliations:** 1 Department of Orthopaedic Surgery, Sapporo Medical University School of Medicine, Sapporo, Japan; 2 Sapporo Sports Clinic, Sapporo, Japan; 3 Department of Orthopaedic Surgery, Sunagawa City Medical Center, Sunagawa, Japan; 4 Second Division of Physical Therapy, Sapporo Medical University School of Health Sciences, Sapporo, Hokkaido Japan

**Keywords:** Anterior inferior tibiofibular ligament, Inferior tibiofibular transverse ligament, Interosseous ligament, Magnetic resonance imaging, Posterior inferior tibiofibular ligament, Syndesmosis injury

## Abstract

**Background:**

Transverse ligament and posterior inferior tibiofibular ligament injuries have not been investigated till date because these are difficult to evaluate using standard magnetic resonance imaging. This study aimed to investigate the prevalence of transverse ligament and posterior inferior tibiofibular ligament injuries in syndesmosis-injured ankles using oblique axial magnetic resonance imaging.

**Methods:**

The patients who were diagnosed with syndesmosis injury using magnetic resonance imaging (MRI) within 7 days of the trauma were included. Patients with concomitant fractures were excluded. A total of 34 patients (1 woman and 33 men) with an average age of 22 years (range, 14–64 years) were included. The anterior inferior tibiofibular, interosseous, transverse, and posterior inferior tibiofibular ligaments were classified as intact, partial tear, or complete tear using usual axial and oblique axial MRIs.

**Results:**

There were 8 (23.5%) ankles with an intact, 21 (61.8%) ankles with a partially torn, and 5 (14.7%) ankles with a complete tear of transverse ligament. There were 20 (58.8%) ankles with an intact, 12 (35.3%) ankles with a partially torn, and 2 (5.9%) ankles with a complete tear of posterior inferior tibiofibular ligament. Overall, 50% of the transverse ligament injuries occurred without posterior inferior tibiofibular ligament involvement.

**Conclusions:**

The oblique axial magnetic resonance imaging scan revealed that the prevalence of transverse ligament and posterior inferior tibiofibular ligament injuries in syndesmosis-injured ankles were 76.5 and 41.2%, respectively.

**Supplementary Information:**

The online version contains supplementary material available at 10.1186/s12891-022-05220-0.

## Background

**Fig. 1:**
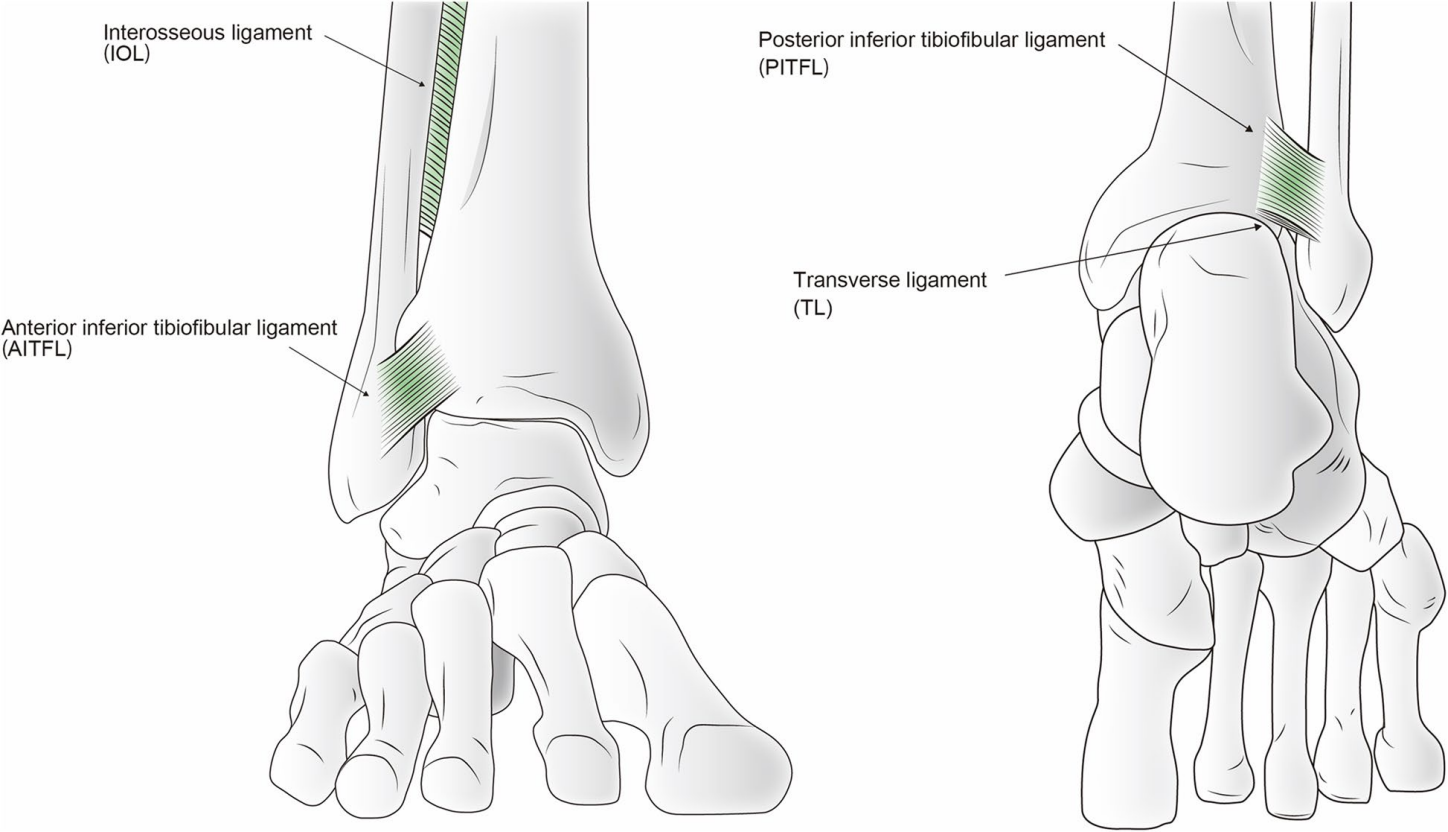
A schema of the anterior and posterior aspects of the ankle joint showing the anterior inferior tibiofibular ligament (AITFL), interosseous ligament (IOL), inferior tibiofibular transverse ligament (TL), and posterior inferior tibiofibular ligament (PITFL)

Syndesmosis injuries of the ankle are often associated with sports injuries and fractures of the ankle. The components of the syndesmosis are the anterior inferior tibiofibular ligament (AITFL), interosseous ligament (IOL), inferior tibiofibular transverse ligament (TL), and posterior inferior tibiofibular ligament (PITFL) (Fig. 1) [1].

Regarding the most appropriate imaging modality for diagnosing syndesmosis injuries, a systematic review reported the following [2]. Conventional radiographs cannot predict syndesmotic injuries reliably. Although computed tomography scans may detect syndesmotic widening, the status of the ligaments cannot be investigated. Magnetic resonance imaging (MRI) has a good sensitivity and specificity for diagnosing syndesmosis injuries. Regarding ultrasound assessment, the sensitivity and specificity are controversial [3, 4].

Syndesmosis injuries can be treated conservatively or operatively depending on the severity of the injury. Grade I syndesmosis injuries with no diastasis are considered as stable, while grade II injuries usually have occult instability with normal alignment of the ankle on standard plain+ radiographs. To diagnose grade II injuries, stress radiographs are usually required. Ankles with grade III injury often have tibiofibular diastasis on standard radiographs [5]. Although conservative treatment is recommended for stable injuries (grade I) and operative treatment is recommended for unstable injuries (grade II and grade III) [5], it is difficult to differentiate between a stable ankle and an ankle with instability, because of severe pain experienced during the stress test. It is important to diagnose grade I or II injuries because their treatment is different. A grade II injury may need surgery to stabilize the syndesmosis. Moreover, in grade II injuries, there are wide range of instability, suggesting that there are several patterns of injury of the ligaments.

Several current classification systems for syndesmosis injuries have been reviewed in the European Society of Sports Traumatology, Knee Surgery, and Arthroscopy-History Ankle and Foot Associates (ESSKA-AFAS) consensus guidelines [5]. Most studies define Grade I injury as an isolated AITFL injury and grade III injury as complete syndesmosis injury including PITFL injury, with respect to the ligament injury pattern. However, grade II injury, which includes AITFL injury with or without IOL injury, interosseous membrane injury, deltoid ligament injury, medial malleolus fracture, and PITFL injury, has not been clearly defined. Therefore, the ligament injury pattern of grade II injury of the syndesmosis is controversial.

Few studies have investigated the rate of TL injuries, because it is difficult to assess and differentiate TL and PITFL injuries using MRI and arthroscopy [6]. Although the importance of the TL in providing stability to the syndesmosis was demonstrated in a cadaveric biomechanical study [7], not much literature about the TL exists. MRI has good sensitivity and specificity for evaluating syndesmotic injuries [8]. However, the TL and PITFL are not clearly visualized by the standard axial plane MRI scans [6, 9]. As the AITFL, TL, and PITFL run obliquely, axial plane MRI may show partly interrupted ligaments, resulting in a false-positive diagnosis of partial injuries of the ligaments [6, 9]. Therefore, we used an oblique image plane MRI to assess AITFL, TL, and PITFL injuries.

This study aimed to investigate the prevalence of TL and PITFL injuries in syndesmosis-injured ankles. We hypothesized that TL or PITFL injury may occur in the absence of injury.

to other syndesmotic ligaments.

## Methods

### Design

This study is a retrospective screening of institutional databases and was designed to include patients who complained of pain and were diagnosed with syndesmosis injury clinically by an orthopedic surgeon and with MRI assessment within 7 days of the trauma, between January 2018 and June 2019. Patients with concomitant fractures or a history of previous ankle surgery were excluded from the study. The study comprised 34 patients (1 woman and 33 men) and the average age of the patients was 22 years (range, 14–64 years).

### Magnetic resonance imaging

**Fig. 2:**
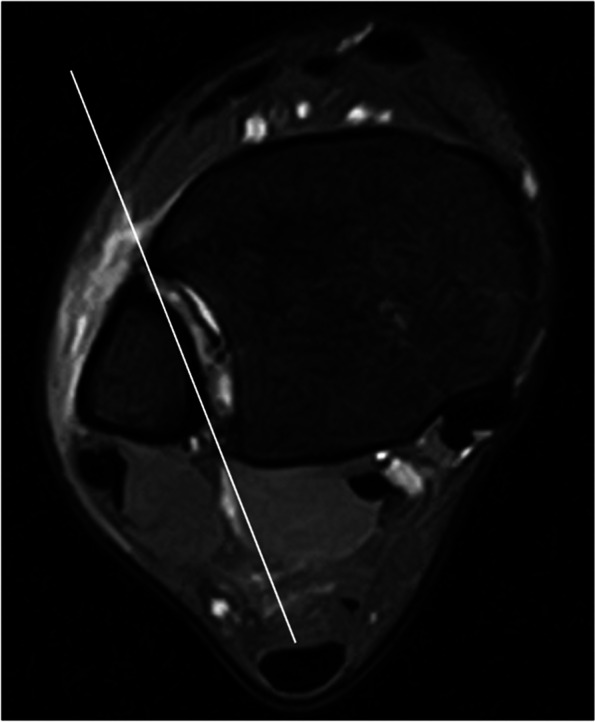
Oblique axial slice for demonstrating ligaments in their full length. In the sagittal view, the oblique plane runs parallel to a line

**Fig. 3:**
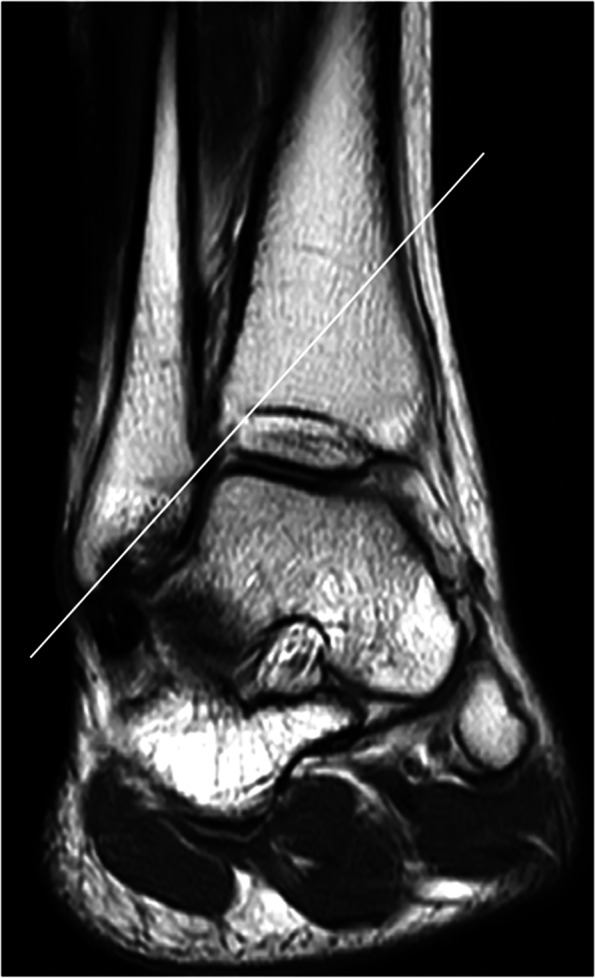
Oblique axial slice for demonstrating ligaments in their full length. In the coronal view, the angle between the oblique plane and the tibial plafond is approximately 45

**Fig. 4:**
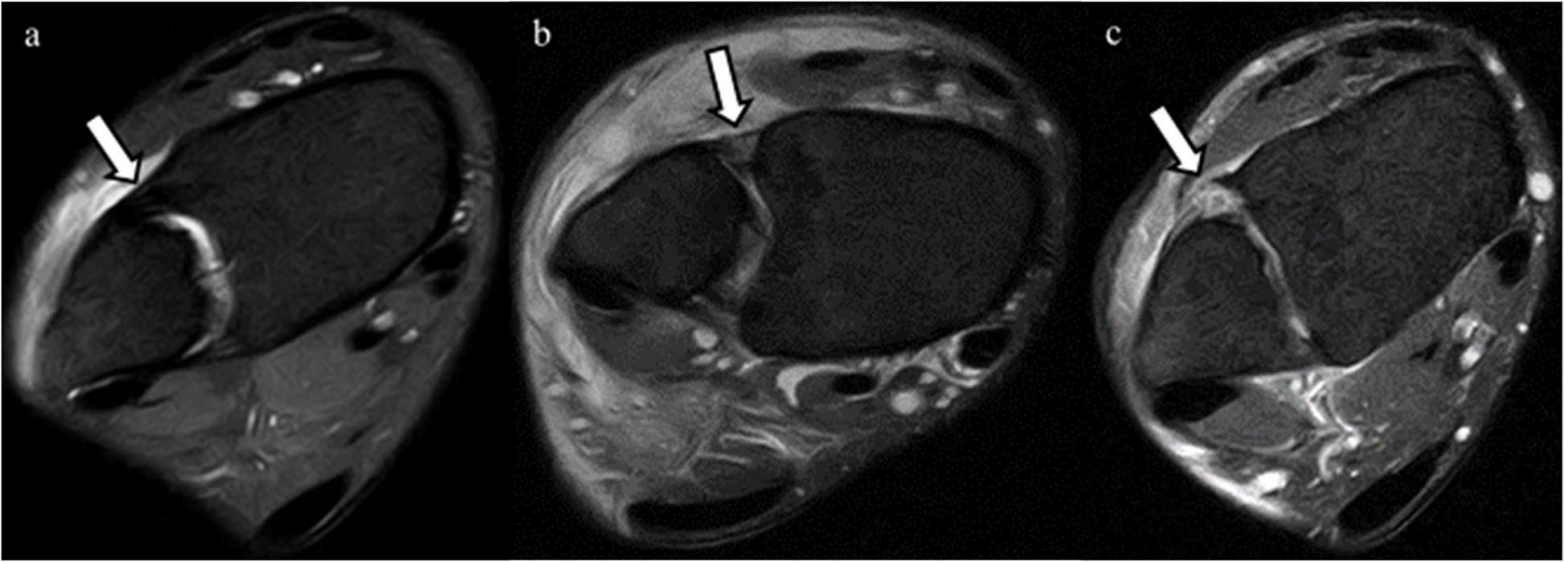
Anterior inferior tibiofibular ligament (AITFL) in oblique axial proton-density fat saturation MRI. The arrows in the figures indicate the AITFL. **a** The intact AITFL. **b** Partial tear of the AITFL, with hyperintensity of the ligament. **c** Complete tear of the AITFL, showing complete discontinuity

**Fig. 5:**
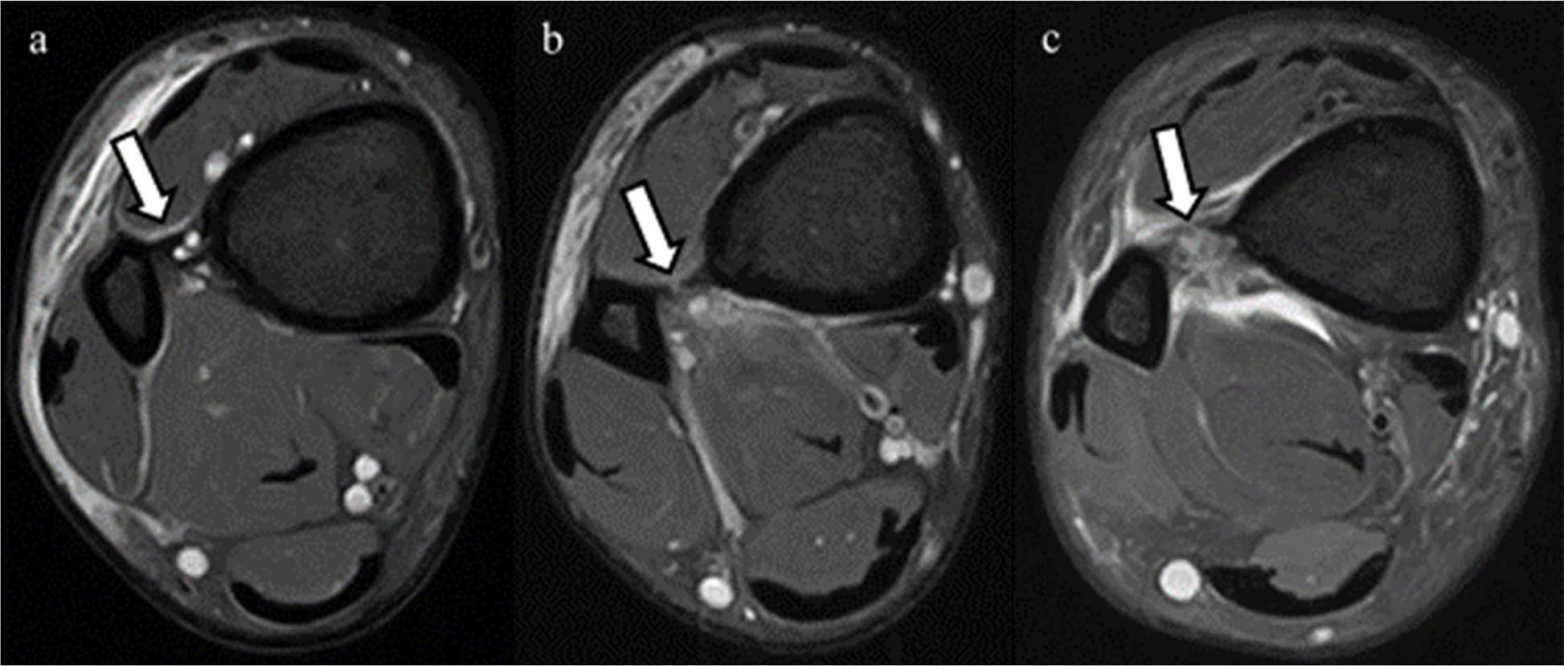
Interosseous ligament (IL) in oblique axial proton-density fat saturation MRI. The arrows in the figures indicate IL. **a** The intact IL. **b** Partial tear of the IL, with complete discontinuity in this slice, but continuity is visible in the other slices. **c** Complete tear of the IL, showing complete discontinuity

**Fig. 6:**
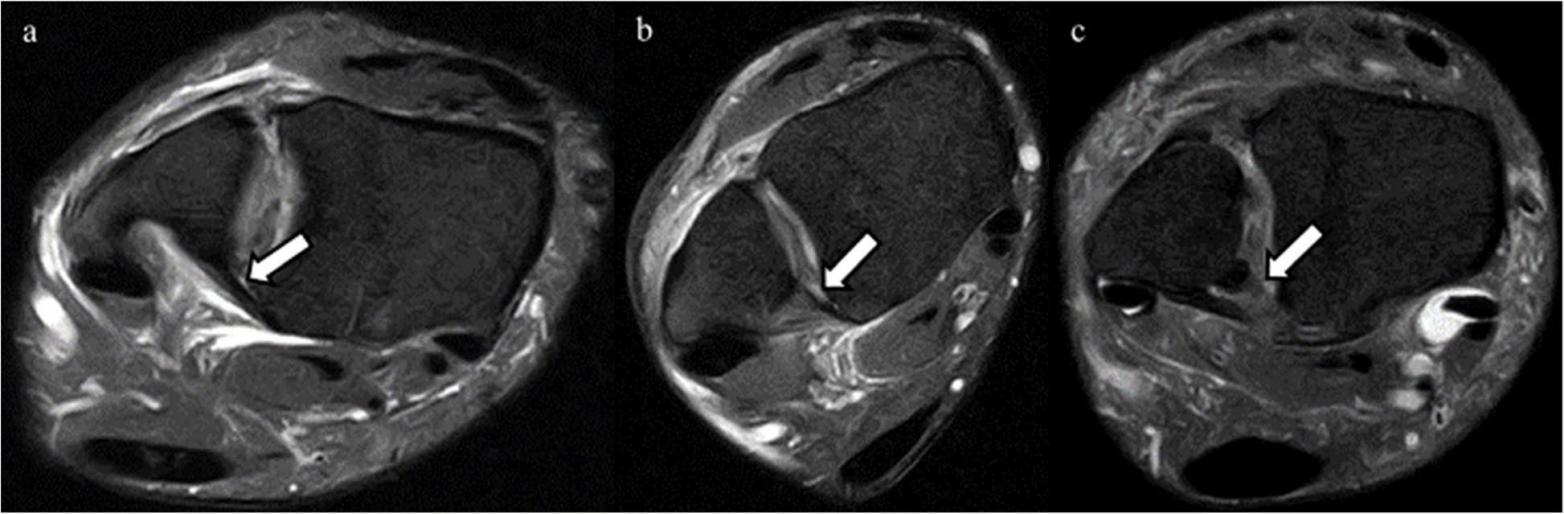
Transverse ligament (TL) in oblique axial proton-density fat saturation MRI. The arrows in the figures indicate TL. **a** The intact TL. **b** Partial tear of the TL, with hyperintensity of the ligament. **c** Complete tear of the TL, showing complete discontinuity

**Fig. 7:**
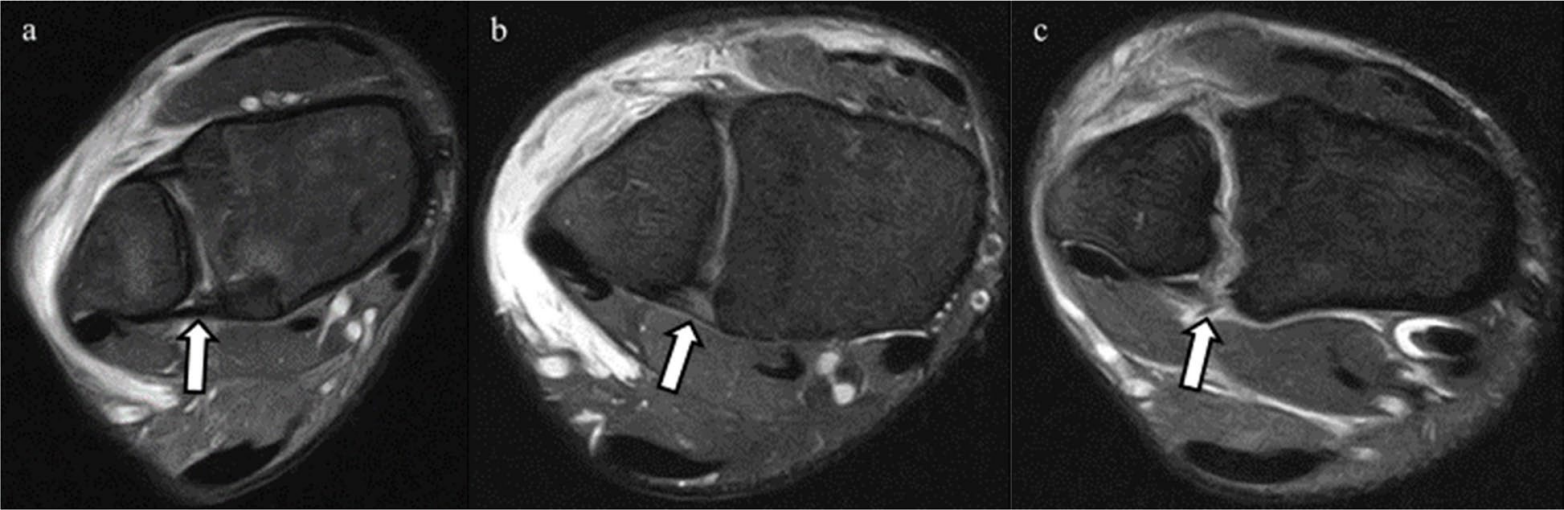
Posterior inferior tibiofibular ligament (PITFL) in oblique axial proton-density fat saturation MRI. The arrows in the figures indicate PITFL. **a** The intact PITFL. **b** Partial tear of the PITFL, with hyperintensity of the ligament. **c** Complete tear of the PITFL, showing complete discontinuity

MRIs were performed within 7 days after trauma using a 1.5-T ECHELON RX MRI unit (Hitachi Healthcare, Tokyo, Japan). All ankles were neutrally positioned with the patient in supine position. The MRI assessment consisted of axial, sagittal, coronal, and oblique axial slice proton-density (PD) fat saturation (repetition time/echo time, 1110 ms/33 ms; DE Pulse ON; matrix, 256 × 256; echo train length, 5; voxel size, 3.0 mm; slice thickness, 3.3 mm) fast spin-echo. To evaluate injuries of the AITFL, TL, and PITFL, an oblique axial slice demonstrating the full lengths of these ligaments was obtained according to a previous report with some modifications [9]. In the sagittal view, the direction of the oblique plane ran parallel to a line along the inferior lateral margin of the tibia in an anterior to posterior direction (Fig. 2). In the coronal view, the angle between the oblique plane and the tibial plafond was adjusted to approximately 45° to depict the full lengths of the ligaments (Fig. 3). The status of the IOL was evaluated using axial slice MRI approximately 25 mm above the tibial plafond. All MRI scans were independently evaluated by board certified orthopedic surgeons. The AITFL, IOL, TL, or PITFL was classified on the basis of morphological features and signal intensity of the ligaments as intact, partial tear, or complete tear (Figs. 4, 5, 6 and 7). An uninjured ligament without discontinuity or fluid signal was considered intact. A partial tear was characterized by a fluid signal transecting some of the ligament fibers or hyperintensity of the ligament and a complete tear was characterized by complete discontinuity of the ligament.

### Statistical analyses

All statistical analyses were performed using SPSS ver. 23.0 (IBM Corp., Armonk, NY). For intraobserver reliability, the first observer performed MRI evaluations twice, with a period of 4 weeks between the first and second MRI evaluations. For interobserver reliability, the second observer evaluated the MRI once. The intra- and interobserver agreements for the evaluation of ligament status via oblique axial MRI were calculated using the kappa agreement score (0.00–0.20 poor, 0.21–0.40 fair, 0.41–0.60 moderate, 0.61–0.80 good, 0.81–0.90 very good, 0.91–1.00 extra good).

## Results

The status of the ligaments on evaluation of the axial and oblique axial MRI scans is summarized in Table 1. AITFL was completely torn in 26 (76.5%) ankles and partially torn in 8 (23.5%) ankles. None of the AITFL ligaments in this cohort were intact following the injury. IOL was completely torn in 8 (23.5%) ankles, partially torn in 16 (48.1%) ankles, and was intact in 10 (29.4%) ankles. TL was completely torn in 5 (14.7%) ankles, partially torn in 21 (61.8%) ankles, and remained intact in 8 (23.5%) ankles. PITFL was completely torn in 2 (5.9%) ankles, partially torn in 12 (35.3%) ankles, and was intact in 20 (58.8%) ankles.

**Table 1:** Prevalences of AITFL^a^, IOL^b^, TL^c^, and PITFL^d^ injuries

	AITFL	IOL	TL	PITFL
Intact	0 (0.00%)	10 (29.4%)	8 (23.5%)	20 (58.8%)
Partial injury	8 (23.5%)	16 (48.1%)	21 (61.8%)	12 (35.3%)
Complete injury	26 (76.5%)	8 (23.5%)	5 (14.7%)	2 (5.9%)

^a^*AITFL* Anterior inferior tibiofibular ligament

^b^*IOL* Interosseous ligament

^c^*TL* Inferior tibiofibular transverse ligament

^d^*PITFL* Posterior inferior tibiofibular ligament

PITFL involvement was observed with 50% of all TL injuries. An additional table file shows the patterns of the injured ligaments in greater detail [see Additional file 1].

The kappa values for intra- and interobserver agreements for the evaluation of ligament status using oblique axial MRI scans were between 0.77–0.92, indicating good, very good, and extra good reliability (Table 2).

**Table 2:** Kappa values of intra- and interobserver agreements for evaluation of ligament status on oblique axial MRI

	AITFL^a^	IOL^b^	TL^c^	PITFL^d^
Intraobserver	0.922	0.815	0.886	0.834
Interobserver	0.837	0.770	0.891	0.825

^a^*AITFL* Anterior inferior tibiofibular ligament

^b^*IOL* Interosseous ligament

^c^*TL* Inferior tibiofibular transverse ligament

^d^*PITFL* Posterior inferior tibiofibular ligament

## Discussion

In this study, the status of the ligaments in syndesmosis-injured ankles without concomitant fractures were investigated using oblique axial MRI. The prevalence of TL and PITFL injuries in syndesmosis injured ankles was 76.5 and 41.2%, respectively. Moreover, injury pattern analysis demonstrated that 50% of TL injuries occurred without PITFL injury.

Previous studies reported that the prevalence of PITFL injury by MRI evaluation was between 8.6 and 97.2% (Table 3) [6, 8, 10, 11]. However, when interpreting the data of these papers, we should consider the difficulty in differentiating TF and PITFL injury using MRI scans, the presence of false-positive cases, and the rate of concomitant fractures. Oae [6] and Takao et al. [8] evaluated PITFL injuries based on morphological criteria such as discontinuity of ligament, decreased tension, an abnormal course of the ligament, and a wavy or curved ligament contour. These criteria seem to evaluate only complete injury of the ligament, which may be a reason for the relatively low rate of PITFL injuries reported by these studies. Contrarily, Park [10] and Randell et al. [11] reported a high prevalence of PITFL injuries. In the former study, MRI assessments were performed by 22 radiologists and PITFL injury was recorded without any differentiation between partial and complete injuries to each individual ligament. In the study by Park et al. [10], PITFL injury was classified as intact, partial tear, or complete tear. In addition to the method of evaluating PITFL injury, the rate of concomitant fractures may affect the rate of PITFL injury, since a higher rate of concomitant fracture may lead to a higher rate of PITFL injury (Table 3). Moreover, Oae [6] and Hermans [9] reported that evaluation using standard axial MRI included false-positive cases. Therefore, considering our method to evaluate ligament status without fractures, we believe the low rate of PITFL injury reported in this study is reasonable.

**Table 3:** Prevalences of concomitant fractures and PITFL injuries evaluated by MRI or radiography in the previous papers and the present study

	N	Concomitant fracture	Partial or Complete PITFL^a^ injury	Complete PITFL injury
Park [10]	74	100.0%	97.2%	25.7%
Randell [11]	164	above 15.7%	67.7%	NA
Takao [8]	52	63.5%	NA	19.2%
Oae [6]	58	39.7%	NA	8.6%
This study	34	0.0%	41.2%	5.9%

^a^*PITFL* Posterior inferior tibiofibular ligament

Although conservative treatment is recommended for stable injuries (grade I) and operative treatment is recommended for unstable injuries (grades II and III), differentiating between a stable ankle and an ankle with occult instability on stress radiography is challenging. Moreover, beyond 10 days after injury, accurate assessment of the stability of the syndesmosis may be difficult [12, 13]. Evaluation of the ligament injury pattern of grade II injury using an MRI scan remains controversial [5]. In addition, an important clinical obstacle to the treatment of syndesmosis injury is the unexpected clinical outcome associated with chronic ankle instability or pain despite a diagnosis of grade I injury [14]. The difficulty of distinguishing between grade I and II injuries can lead to misclassification and incorrect treatment of grade II injuries, which may be one of the reasons for the unexpected clinical outcomes of syndesmosis injuries. Therefore, we believe that accurate and early diagnosis of grade II injuries could solve clinical problems and investigation of the injury pattern is important for the treatment of syndesmosis injury. In this study, we demonstrated that TL injury without PITFL involvement may be an important injury pattern of grade II injury, which may provide new information for the assessment of syndesmosis injury.

Although several studies describe TL via MRI evaluation [15–17], few studies have reported the clinical prevalence of TL injury. Park et al. [10] reported that a complete tear of the PITFL was the most reliable predictor of instability of the syndesmosis evaluated by arthroscopy; however, they did not investigate the TL status. In a cadaveric biomechanical study, the percentage resistance to 2 mm diastasis was measured for the four ligaments of the syndesmosis [7]. The percentage resistances were 35% for the AITFL, 22% for the IOL, 9% for the PITFL, and 33% for the deep PITFL. The results indicate the biomechanical importance of TL injury without PITFL involvement for the stability of syndesmosis. Therefore, we believe that the injury of TL without PITFL involvement may provide significant information to elucidate the pathological status of syndesmosis injury with occult instability. Although the AITFL, IOL (TL), and PITFL are the first, second, and third most frequently injured ligaments, respectively, there were untypical ankles with AITFL, IOL, and PITFL injuries without TL injury or with AITFL, TL, and PITFL injuries. Such injuries patterns may affect the grading of syndesmosis injuries.

This study has several limitations. First, we evaluated the ligaments using MRI scans alone without arthroscopic findings. Second, this was a retrospective observational study. Third, syndesmotic stability was not evaluated. Although stress radiographs were obtained when possible, some patients could not endure pain during the stress test. Fourth, the study comprised mostly of male patients, which resulted in a selection bias. Fifth, our MRIs were PD images using a 1.5-Tesla resonance instead of T2 weighted images using 3.0 T. MRI assessment was performed only by orthopedic surgeons. The evaluation could be more accurate if T2 weighted images with a 3.0-Tesla resonance or isovolumetric voxel technique was used and the assessment was performed by a musculoskeletal radiologist.

MRI scans in the standard axial plane were reported to show partly interrupted ligaments, leading to false-positive diagnosis of partial ligament injuries [6, 9]. Contrarily, in this study, the kappa agreement score for evaluating syndesmosis injury using oblique axial MRI scans was above 0.77. Based on these results, we believe that oblique axial MRI enables accurate evaluation of the AITFL, TL, and PITFL. Moreover, this is the first study to evaluate TL status, which is an important stabilizer of syndesmosis [7]. We believe that these data are useful for analyzing the pathology of syndesmotic injuries.

## Conclusions

The prevalence of TL and PITFL injuries in syndesmosis-injured ankles without fracture, which were evaluated by oblique axial MRI, were 76.5 and 41.2%, respectively. Additionally, 50% of TL injuries occurred without PITFL injury. This injury pattern may provide new information for elucidating the pathological status of syndesmotic injuries.

## Supplementary Information

**Additional file 1.**

## Data Availability

Not applicable.

## Abbreviations

AITFL: Anterior inferior tibiofibular ligament
IOL: Interosseous ligament
TL: Transverse ligament
MRI: Magnetic resonance imaging
PITFL: Posterior inferior tibiofibular ligament
ESSKA-AFAS: European Society of Sports Traumatology, Knee Surgery, and Arthroscopy History Ankle and Foot Associates
