# Supplementary material for: The prevalence of posterior inferior tibiofibular ligament and inferior tibiofibular transverse ligament injuries in syndesmosis-injured ankles evaluated by oblique axial magnetic resonance imaging: a retrospective study
Source: BMC Musculoskelet Disord. 2022 Mar 18;23:264. doi: 10.1186/s12891-022-05220-0 (PMC8932331; doi:10.1186/s12891-022-05220-0)
Supplement: Supplementary file 1 — Additional file 1. [file 12891_2022_5220_MOESM1_ESM.docx]

**Additional Files**

The pattern of the injured ligaments

| Injured ligaments | | | | Number (%) |
| --- | --- | --- | --- | --- |
| AITFL |  |  |  | 3 (8.8) |
| AITFL | +IOL |  |  | 4 (11.8) |
| AITFL |  | +TL |  | 4 (11.8) |
| AITFL | +IOL | +TL |  | 9 (26.5) |
| AITFL | +IOL |  | +PITFL | 1 (2.9) |
| AITFL |  | +TL | +PITFL | 3 (8.8) |
| AITFL | +IOL | +TL | +PITFL | 10 (29.4) |

AITFL: anterior inferior tibiofibular ligament

IOL: interosseous ligament

TL: inferior tibiofibular transverse ligament

PITFL: posterior inferior tibiofibular ligament
